# Associations of weather and air pollution with objective physical activity and sedentary time before and after bariatric surgery: a secondary analysis of a prospective cohort study

**DOI:** 10.1088/2515-7620/ad64b2

**Published:** 2024-07-31

**Authors:** Aurélie Baillot, Paquito Bernard, Jmii Nejm Eddine, J Graham Thomas, Leah M Schumacher, Pavlos K Papasavas, Sivamainthan Vithiananthan, Daniel Jones, Dale S Bond

**Affiliations:** 1 École Interdisciplinaire de santé, Université du Québec en Outaouais, Gatineau, Québec, Canada; 2 Institut du savoir de l’hôpital Montfort-recherche, Ottawa, Ontario, Canada; 3 Centre de Recherche en Médecine Psychosociale, Centre Intégré de Santé et Services Sociaux de l’Outaouais, Gatineau, Québec, Canada; 4 Department of Physical Activity Sciences, Université du Québec à Montréal, Montréal, Québec, Canada; 5 Institut universitaire en santé mentale de Montréal, Montréal, Québec, Canada; 6 Department of Natural Sciences, Université du Québec en Outaouais, Gatineau, Québec, Canada; 7 Department of Psychiatry and Human Behavior, Weight Control and Diabetes Research Center, The Miriam Hospital/Brown Alpert Medical School, Providence, Rhode Island, United States of America; 8 Department of Social and Behavioral Sciences/Center for Obesity Research and Education, College of Public Health, Temple University, Philadelphia, PA, United States of America; 9 Department of Surgery, Hartford Hospital/Hartford Healthcare, Hartford CT, United States of America; 10 Department of Surgery, Cambridge Health Alliance, Cambridge, MA, 02139, United States of America; 11 Department of Surgery, Rutgers New Jersey Medical School, Newark, NJ, 07103, United States of America; 12 Department of Research, Hartford Hospital/Hartford Healthcare, Hartford CT, United States of America

**Keywords:** weather, air pollution, physical activity, obesity

## Abstract

**Purpose.:**

Identifying factors that influence moderate-to-vigorous intensity physical activity (MVPA) and sedentary time in metabolic and bariatric surgery (MBS) patients is necessary to inform the development of interventions. Weather/environmental factors may be especially important considering rapid climate change and the vulnerability of people with obesity to heat and pollution. Our study aimed to examine the associations of weather (maximal, average and Wet Bulb Globe Temperatures), and air pollution indices (air quality index [AQI]) with daily physical activity (PA) of both light (LPA) and MVPA and sedentary time before and after MBS.

**Materials and methods.:**

Participants (n = 77) wore an accelerometer at pre- and 3, 6, and 12-months post-MBS to assess LPA/MVPA/ sedentary time (min/d). These data were combined with participants’ local (Boston, MA or Providence, RI, USA) daily weather and AQI data (extracted from federal weather and environmental websites).

**Results.:**

Multilevel generalized additive models showed inverted U-shaped associations between weather indices and MVPA, with a marked reduction in MVPA for daily maximal temperatures ≽20 °C. Sensitivity analysis showed a less marked decrease of MVPA (min/d) during higher temperatures after versus before MBS. Both MVPA before and after MBS and sedentary time before MBS were negatively impacted by higher AQI levels.

**Conclusion.:**

This study is the first to show that weather and air pollution indices, even in locations with good AQI and moderate temperatures, are related to variability in activity behaviors, particularly MVPA, during pre- and post-MBS. Weather/environmental conditions should be considered in MVPA prescription/strategies for adults who have undergone MBS.

## Introduction

Low physical activity and excessive sedentary time are common among patients who undergo metabolic and bariatric surgery (MBS) [1, 2]. Because regular physical activity can help optimize MBS outcomes [3], promoting higher physical activity and lower sedentary time levels is an important part of multidisciplinary MBS care [4].

To promote a more active lifestyle among MBS patients, it is critical to identify barriers and facilitators of physical activity and sedentary time change. However, most research has focused on individual-level factors and neglected environmental factors, especially those related to the physical environment. According to the socioecological model [5], factors that influence behavior should be considered from multiple levels of influence including environmental, especially in the context of global climate change and warming.

Two environmental factors that may influence physical activity and sedentary time are shifts in weather patterns and air pollution [6]. Recent reviews involving participants across multiple countries and continents show that physical activity levels are greater during higher outdoor temperatures whereas sedentary time is greater during lower outdoor temperatures [7–9]. Studies also show that Wet Bulb Globe Temperature (WBGT), which takes into account the effects of radiation, humidity, temperature and wind speed on perception of temperature, is negatively associated with daily steps [10], active transport [11], and outdoor occupational related physical activity [12]. Similarly, higher air pollution has been linked to lower physical activity [13–15]. One US study showed that increased fine particle matter related to a 16%–35% increase in the odds of leisure-time physical inactivity [14]. A study conducted in China also suggested that fine particule matter was associated with an increase in daily sedentary time [16]. Another study in China found an increase of one standard deviation in air pollution in China, measured via the Air Quality Index (AQI, which combines carbon monoxide, nitrogen dioxide, ozone, sulphur dioxide, particulate matter measures), related to a 7.3 h increase in weekly sedentary time [15].

Among adults with obesity, weather conditions (e.g., higher precipitation and temperature) are related to lower physical activity [17, 18]. Additionally, shifts in weather patterns and air pollution appear to have more detrimental effects on physical activity in people with obesity relative to those with a healthy weight. For example, one study found that adults with obesity are more vulnerable to heat stress, and that this vulnerability contributes to larger physical activity reductions [19].

However, no study has investigated associations of weather and air pollution with physical activity and sedentary time before and after MBS. MBS is the most effective weight loss intervention for individuals with severe obesity. It provides an ideal model to understand how temperature and pollution influence engagement in physical activity and sedentary behaviors before and during rapid and substantial weight loss. Our use of this model in this context is not only highly novel but important because of the potential to generate hypotheses and future research that examines about how and why different environmental exposures might differentially impact physical activity in the context of obesity and significant weight reduction. For example, it is possible that physiological changes after MBS could help reduce the degree to which outdoor temperatures and air pollution are a barrier to physical activity. Indeed, MBS may help increase tolerance to higher outdoor temperatures (via reduced adipose tissue and improved thermoregulation) and air pollution (via increased respiratory function). This is an important knowledge gap in light of current and future climate changes and their expected impact on health and behavior.

Thus, we aimed to explore associations of objective daily weather (maximal and averaged temperature, WBGT) and air pollution (i.e., AQI) with device-measured physical activity (i.e., light-intensity physical activity [LPA], and moderate-to-vigorous intensity physical activity [MVPA]) and sedentary time before and after MBS. We hypothesized that higher daily weather temperatures, WBGT and AQI levels would be associated with lower physical activity, especially MVPA, and greater sedentary time.

## Methods

### Participants

This study involves analysis of data from a prospective cohort study that evaluated multiple behavioral and psychosocial predictors of MBS outcomes. To be initially eligible, participants had to have a BMI ≽ 35 kg m^−2^, be ≽21 years old, and be scheduled to undergo Roux-en-Y gastric bypass (RYGB) or sleeve gastrectomy (SG) at one of two university-based hospitals in the Northeastern US (Boston/Providence). A total of 92 participants consented to participate at baseline, 77 of whom provided valid accelerometry data at baseline. The number of participants who met valid accelerometer wear time requirements at 3, 6, and 12 months post-surgery follow-up was 60 (78%), 54 (70%) and 44 (57%), respectively (Supplemental figure 1 for flow chart).

### Procedure

Aspects of the parent study protocol that are relevant to the present analyses are described below; the full study protocol is published elsewhere [20]. The STrengthening Reporting of OBservational studies in Epidemiology checklist for observational studies was used in this study (Supplemental table 1).

Participants were recruited on a rolling basis between 5/1/2016 and 4/3/2018 at a clinic visit occurring 3–8 weeks before their surgery. Participants deemed initially eligible after telephone screening completed an in-person screen/baseline assessment at the MBS clinic or affiliated research center. Participants were not included if they were enrolled in another weight loss program or related behavioral treatment outside standard surgical care. During this visit, participants provided informed consent, had their height and weight measured, completed a demographic questionnaire, and received an accelerometer to wear for 10 days before surgery. Participants wore the accelerometer again at 3, 6, and 12 months after surgery. For the current study, daily accelerometer data from participants were combined with daily weather data using each participant’s city address/location and accelerometer wear time dates. Measures and data sources are detailed below. The parent study was approved by the institutional review boards of The Miriam Hospital (TMH) in Providence, RI, USA and Beth Israel Deaconess Medical center (BIDMC) in Boston, MA. The study was registered at www.clinicaltrials.gov (NCT02777177).

### Measures

Objectively-measured physical activity and sedentary time. Participants’ physical activity and sedentary time were recorded using an ActiGraph GT9X Link wrist-worn triaxial accelerometer. Valid wear time was defined as ≽7 days with ≽10 h of wear per day. Sleep periods were identified and removed from analysis using the Cole-Kripke and Tudor-Locke algorithms within the ActiLife Version 6.13.3 software (ActiGraph, LLC, Pensacola, FL, USA). Nonwear periods, defined as ≽90 min without movement using vector magnitude counts and with allowance of interruptions of ≼2 min of nonzero counts, were also identified and removed [21, 22]. Vector magnitude counts per minute (cpm) thresholds that have been shown to minimize the mean difference between estimates of sedentary time and MVPA when using wrist-versus hip-based ActiGraph accelerometers were used to estimate minutes/day of sedentary time (<2000 cpm), LPA(≽2000 <7500 cpm) and MVPA (≽7500 cpm) [23].

### Independent variables: weather indices and air pollution data

Daily physical activity and sedentary time data across assessments were combined with local daily weather data according to participants city localization (Boston/Providence) and accelerometer wear time dates. Averaged and maximal temperatures (°C), precipitation and snow fall (cm) were obtained from the USW00014739 and USW00014765 weather stations, which were the closest to Boston and Providence. AQI data were acquired from monitoring stations which are present in core-based statistical areas of both Boston-Cambridge-Newton and Providence-Warwick. AQI is an index combining carbon monoxide, nitrogen dioxide, ozone, sulphur dioxide, and particulate matter measures [24]. Weather data and AQI were extracted from the websites of the National Center of Environment Information of the National Oceanic and Atmospheric Administration, and the Environmental Protection Agency, respectively. Higher AQI value are associated with a greater level of air pollution. An AQI >50, >100, > 200, and > 300 were considered as moderate, unhealthy for sensitive groups, unhealthy, very unhealthy, and hazardous, respectively [25]. There is a high health risk to perform MVPA for unfit or non-acclimatized adults with WBGT >25.7 °C (78.1°F) [26].

### Covariates

Sociodemographic covariates included gender, age, ethnicity, education level, and BMI.

### Statistical analysis

To investigate the association between environmental and movement related data, the shape of relationships (linear versus non-linear) was first investigated [7, 27, 28]. Consequently, multilevel linear and generalized additive models (GAM) [29] were carried out for each tested association. Participants were nested within their respective recruitment center. These models included movement behaviors (MVPA, LPA, sedentary time) as dependent variables, and time varying covariates (days number, season, weekend day (Yes/No), precipitation, snowfall). To take time autocorrelation into account, lag-1 of dependent and weather variables were also included in models. A cubic spline smoother was used to fit GAMs. Both models were compared using Akaike’s Information Criterion (AIC), with smaller values for the information criterion indexes indicating a better model [30]. Then, the selected model (linear or non-linear) was executed again by including socio-demographic covariables. If a statistically significant association was found in GAMs, a 2-dimensional plot was produced. The packages mgcv, tidyverse, lmer, gtsummary, hydroTSM and weather metrics form the statistical software R (v4.2) were used to prepare, conduct and visualise the models. To examine the potential effect of MBS, a set of sensitivity analyses and visual examination of plots was performed [31]. All selected full models were separately carried out for pre- and post-surgery data. Statistical analyses were performed with a total of accelerometry and environmental combined data of 2577 days (i.e., 4 times of assessment including 7 to 11 days of data collection). This number of timepoint with the same temporal resolution is adequate to detect possible daily associations [32].

## Results

### Participant characteristics

Of the 77 participants included before the surgery, 86% were women, 48% identified as other than White, 37% had earned a college or graduate degree and the mean age was 44.5 ± 11.3 years old. The mean baseline BMI was 45.9 ± 7.6 kg m^−2^. Fifty-seven (80%) and twenty participants (20%) underwent SG and RYGB surgery, respectively. Detailed information on accelerometer data, BMI and data collection season according to the 4 times assessments and seasons are presented in Supplemental table 2.

### Weather indices and air pollution description

The physical activity and sedentary time assessments were performed in Providence and Boston, having had a mean temperature of 12.1 ± 9.1 °C (range: −15.0 °C to 31.7 °C), a WBGT of 8.9 ± 9.4 °C (range: −15.0 °C to 25.0 °C), and an AQI score of 49.6 ± 17.5 (range: 21.0 to 151.0) between June 2016 and April 2019. The air quality during the accelerometer data collection was good (<50), moderate (50–100), and unhealthy (>100) for 62%, 35% and 3% of days, respectively. Also, all accelerometer data were collected during days with a WBGT <25.6 °C (78.1°F), which is below the high-risk threshold to perform MVPA for unfit or non-acclimatized adults.

### Associations of weather indices and air pollution with MVPA

**Figure 1.**
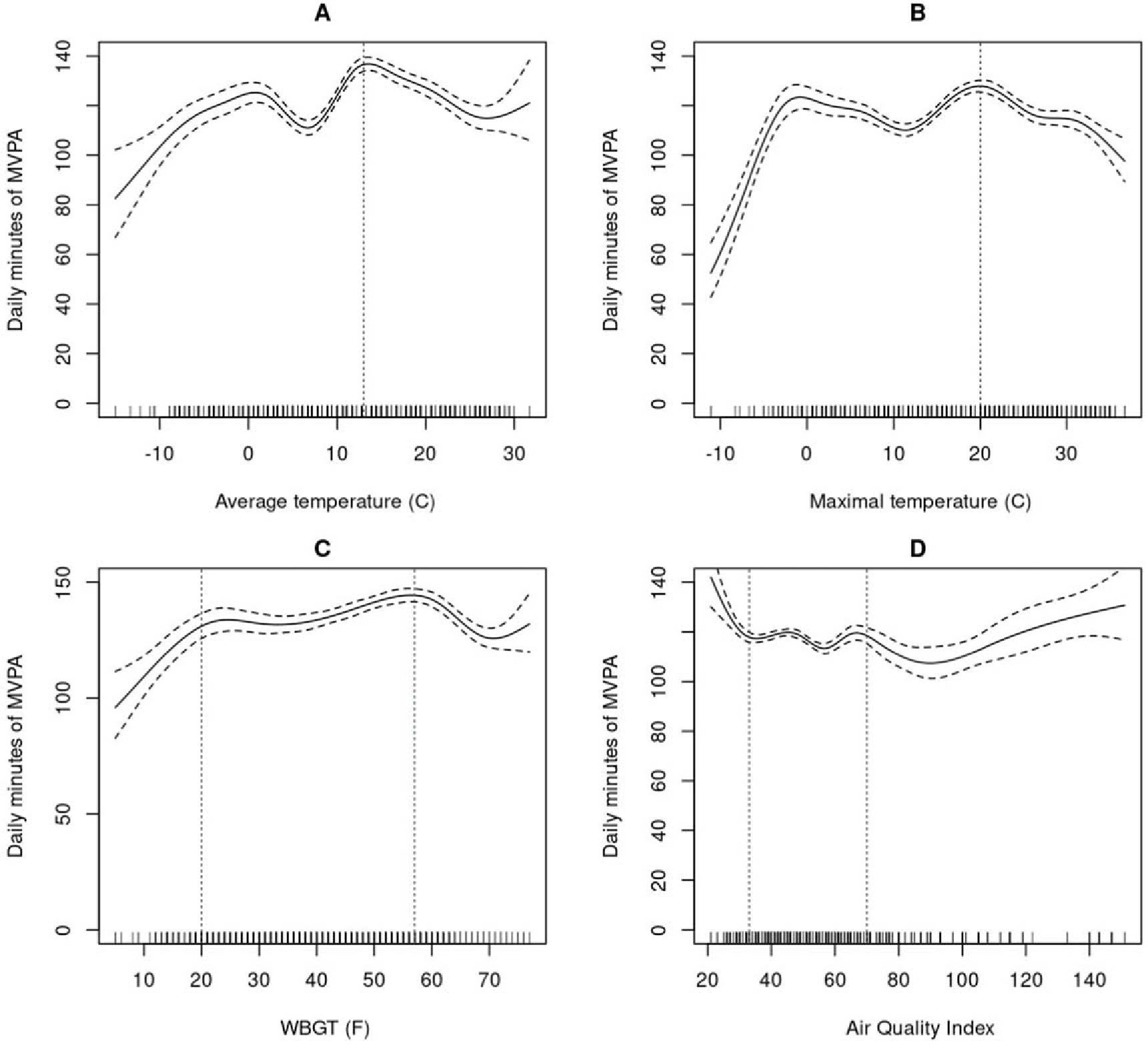
Associations of weather indices(average temperature, maximal temperature, WBGT) and air pollution (Air quality index) with moderate-vigorous intensity physical activity. Note: The lines show the smoothed function from GAM for daily minutes of MVPA, and the shaded area indicates the 95% confidence interval. Each model is adjusted for age, body mass index, gender, days number, season, weekend day (Yes/No), precipitation, snowfall, lag-1 of MVPA and weather variables. Vertical lines are placed based on visual observation.

Non-linear models had a better fit index and showed a statistically significant association between tested weather indices, AQI and MVPA (figure 1, supplemental table 3). Average and maximal temperature were associated with MVPA, p< .001, R^2^ = 0.64, and p<0.001, R^2^ = 0.64, respectively. A visual examination of plots indicated a reduction of MVPA minutes/day after ~13 °C (~55 °F) and ~20 °C (~68 °F) for average and maximal daily temperatures, respectively. An inverted U-shaped was found for association between daily MVPA and WBGT (p < 0.001, R^2^ = 0.63). High MVPA levels occurred between ~ −7 °C (~20 °F) and ~14 °C (~57 °F) WBGT, with a reduction of MVPA below and above these values.

Concerning AQI, a statistically significant association with MVPA (p < 0.001, R^2^ = 0.64) was observed with a non-linear complex shape. A decrease of MVPA minutes/day was observed until ~33 AQI score, followed by a monotonic curve, and an MVPA decrease after ~70 AQI score.

### Associations of weather indices and air pollution with LPA

The shape of the associations of LPA with averaged temperature, maximal temperature, and WBGT were non-linear, but linear with AQI. No statistically significant associations were found between LPA and averaged temperature, maximal temperature, WBGT, AQI in multivariate models (Supplemental table 4).

### Associations of weather indices and air pollution with sedentary time

Non-linear models were selected for tests involving sedentary time. No statistically significant associations of weather and air pollution outcomes with sedentary time minutes/day were found (Supplemental table 5).

### Sensitivity analyses: before and after MBS

**Figure 2.**
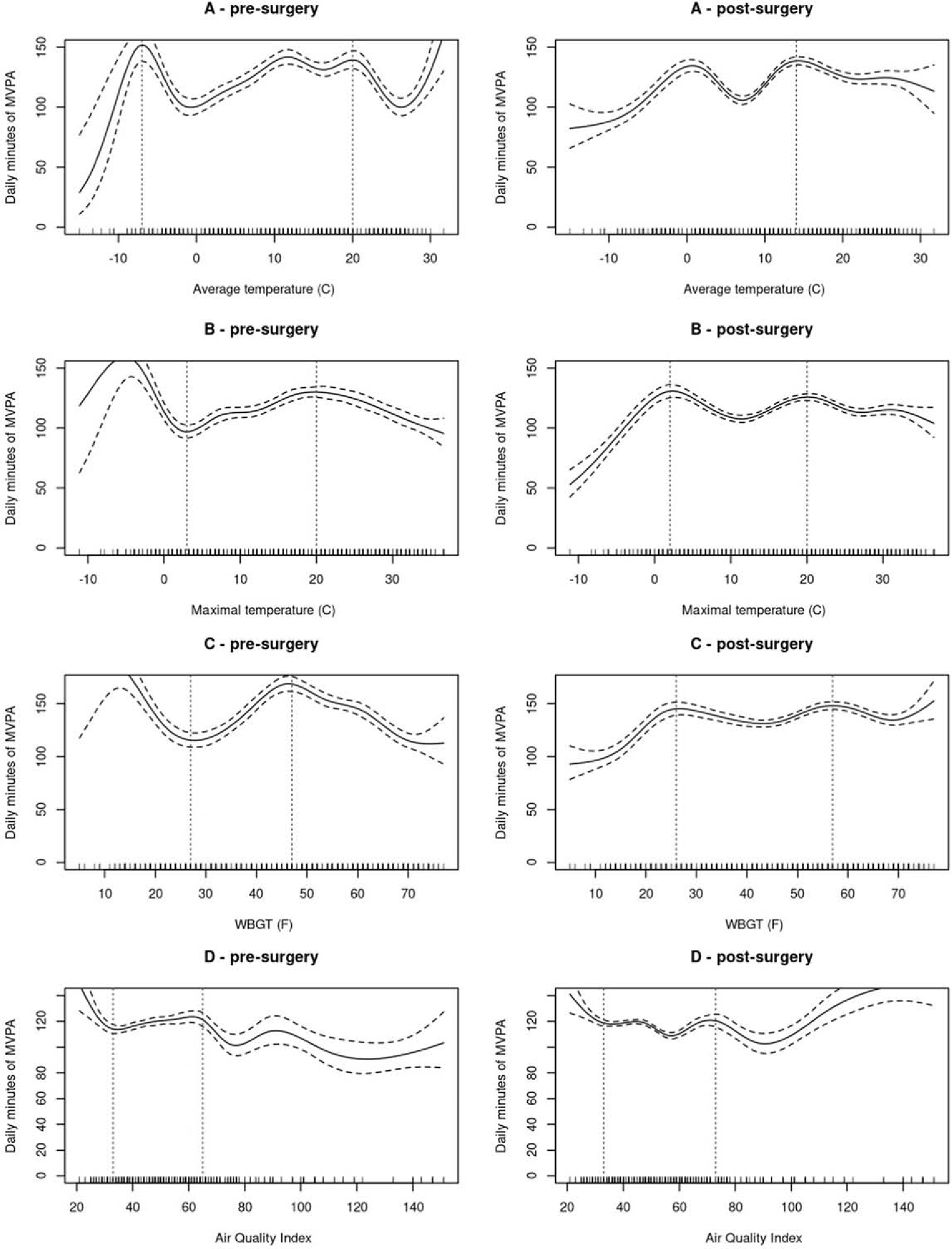
Associations of weather indices(average temperature, maximal temperature, WBGT) and air pollution (Air quality index) with moderate-vigorous intensity physical activity before and after MBS. Note: The lines show the smoothed function from GAM for daily minutes of MVPA, and the shaded area indicates the 95% confidence interval. Each model is adjusted for age, body mass index, gender, days number, season, weekend day (Yes/No), precipitation, snowfall, lag-1 of MVPA and weather variables. Vertical lines are placed based on visual observation.

**Figure 3.**
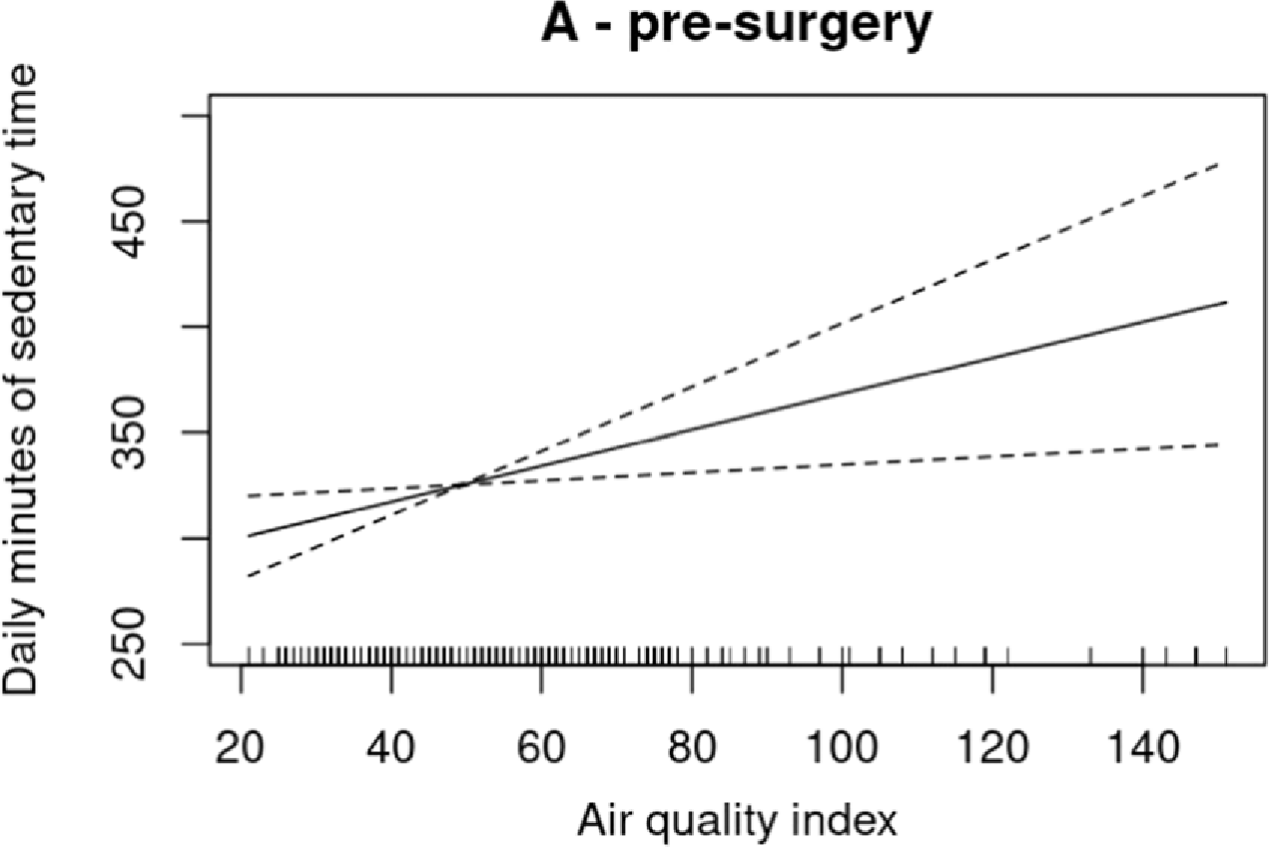
Linear association of air quality index with sedentary time before MBS.

The association shapes for MVPA were globally similar between pre- and post-MBS (figure 2, supplemental table 6). However, a visual examination of plots indicated that a larger reduction in MVPA minutes/day appears before MBS for average temperature at ~20 °C and for WBGT at ~8 °C (~47 °F) compared to after MBS. No significant associations were observed for LPA models (Supplemental table 7). Additionally, a positive linear association was found between sedentary time minutes/day and AQI before MBS (R^2^ = 0.395; p≼.05) (figure 3, supplemental table 8). In addition, as suggested by a reviewer, the associations between weather and AQI data with MVPA across the multiple post-surgery time points were modeled. For descriptive purposes, plots are available in the supplemental figure 2.

## Discussion

Adults who undergo MBS are encouraged to perform regular physical activity to optimize and sustain postoperative weight loss and health improvements. However, many patients are insufficiently active before MBS and struggle to achieve and/or maintain sufficient physical activity levels after MBS [1, 2]. This warrants greater attention to identifying and understanding physical activity barriers in this population. To date, efforts in this regard have mostly focused on individual-level barriers with minimal consideration of how patients’ external environment might inhibit physical activity. Therefore, this study aimed to improve understanding of how variability in objective temperature and air pollution indices relate to objectively-measured daily physical activity and sedentary time on a before and after MBS.

Results showed that MVPA related in a nonlinear fashion to temperature indices. Daily MVPA decreased when maximal temperatures exceeded ~20 °C (~68 °F). The only other similar study found in adults with obesity also showed an inverse U-shape with the probability of leisure-time physical activity markedly decreasing after 36 °C [19].

Similarly, for WBGT, which considers humidity, wind speed, sun angle, and cloud cover along with temperature, the highest MVPA levels were observed between ~ −7 °C (~20 °F) and ~14 °C (~57 °F) WBGT, with MVPA decreasing below and above these values. This WBGT level falls well below the WBGT level of >25.7 °C (78.1 °F) that is associated with high risk to perform MVPA for unfit or non-acclimatized adults. These findings align with previous research suggesting that higher temperatures have a negative effect on physical activity of higher intensities among individuals with obesity [33, 34].

Although overall associations of MVPA and temperature indices were similar between pre- and post-MBS, visual inspection of plots displayed a less marked decrease of MVPA after MBS for average temperature of ~20 °C (~68 °F) and WBGT of ~8 °C (~47 °F) compared to before MBS. While research is needed to substantiate this observation, it is possible that more severe obesity and related higher levels of subcutaneous fat impede heat loss, and that the substantial reduction in subcutaneous fat following MBS may improve thermoregulatory functions and ability to tolerate warmer temperatures while performing MVPA [35].

Similar to studies that examined associations of self-reported physical activity and air pollution [13, 14, 36], our results showed that objectively-measured daily MVPA is negatively associated with air pollution.

Specifically, MVPA levels decreased after ~70 AQI score. Although this value is considered acceptable for the general population, it is considered a risk for some people who are unusually sensitive to air pollution [25]. People with obesity may be more sensitive to air pollutants during MVPA than individuals without obesity given the inflammatory and mechanical (i.e., excess fat on the chest wall and abdominal cavity) effects of obesity decreasing respiratory function and exercise tolerance [37, 38]. Related, we also found that sedentary time before surgery increased with higher levels of air pollution. Taken together, these findings suggest that higher levels of air pollution may contribute to low MVPA among adults who have undergone MBS, and increased sedentary time especially before MBS.

The above findings may have important clinical implications for prescribing and promoting physical activity among adults who have MBS. For example, during warmer temperatures, patients might be advised to perform MVPA during morning hours to limit exposure to hotter temperatures. When exercising outside, patients should be encouraged to hydrate before, during and after their session, wear light-weight clothing, and use wearable cooling wraps to enhance thermoregulatory capacity. Patients might also benefit from exercising outside before 9 AM given that air pollution tends to be higher in homes and workplaces than outside and peaks at 9 AM on average [39, 40].

Given that LPA is also related to health benefits in adults [41], we explored associations of weather and air pollution with LPA. However, we found no statistically significant association of LPA with weather indices, and air pollution, in contrast with some studies reporting association between LPA and outdoor temperature [7, 42]. Nevertheless, evidence on the association between LPA and outdoor temperature is inconsistent, and sparse for air quality [7–9]. Additional research that considers the different forms and contexts of LPA (leisure, transport-related or occupational activity) is needed.

Regarding sedentary time, we found no statistically significant associations with temperature and WBGT, consistent with some studies, but not others [7–9]. In addition, our results showed positive associations between sedentary time and AQI before MBS, but not after MBS. Discrepancies between studies may be explained by factors, such as differences populations, climatic zones studied, statistical model used or covariates considered. Additional studies are required to disentangle discordances, and explore potential confounders. For example, because sedentary time usually occurs indoors, the impact of outdoor environmental factors on sedentary time in MBS patients could be minimal.

## Strengths and limitations

This study is the first to prospectively explore associations of objectively-measured weather and air quality indices and device-measured physical activity and sedentary time before and after MBS. We used a sophisticated multilevel modelling approach that allowed us to capture and analyze nonlinear relationships. While our sample size is relatively small overall and a prior power analysis was not conducted, we note that participants wore a device that recorded minute-by-minute data for ~10 days days at each of 4 assessments. After attrition, the total number of time points is 826, 683, 603, and 465 at pre-surgery baseline and 3, 6, and 12-months post-surgery, respectively. To our knowledge, only two papers have addressed power estimation for time series regression of counts and there is no clear generic method for conducting *a priori* power analysis in such cases [32, 43]. The total number of dependent variables (e.g., death or hospitalizations) and covariates are the dominant factors that increase the risk of low statistical power [32]. By contrast, our dependent variables are behavioral outcomes which allowed for testing associations of daily occurring dependent variables (e.g., physical activity) with each daily occurring environmental independent variable (e.g., AQI). As a result, there is minimal risk of overdispersion or rare events (e.g., daily deaths) that undermine statistical power.

Several limitations should also be acknowledged. Generalizability of results is limited to geographic locations, air quality and weather conditions outside of the Northeastern United States. Previous studies examining associations of weather or air pollution with objective measures of PA show that maximal temperature thresholds at which PA declines differs by US region [9, 19]. Indeed, the current heat adaptation is generally better among adults living in cities within in Southeast, Great Plains and Southwest regions [44]. Additional research is needed to determine whether local temperature related thresholds for physical activity vary by US region among individuals with obesity and those who have achieved weight loss via MBS. Also, future investigations are required to examine whether other obesity treatments (i.e., behavioral interventions, pharmacologic approaches) can modify the impacts of weather or air pollution on physical activity and sedentary related behaviors. Because we did not collect real-time GPS data we could not determine the weather station closest to participants’ location at all times. Other environmental factors not assessed (e.g. availability of cooling strategies or neighborhood walking) may have influenced associations. The identified temperature thresholds were based on visual analysis of plots, and not found with breakpoint statistical models. Indeed, these models can not be carried out with multilevel GAMs in R. Consequently, a threshold identification data-based approach could provide slightly different cut-offs. Finally, accelerometers do not provide information on context and form (leisure, transport-related or occupational activity) of physical activity and sedentary time performed. It is possible that stronger effects might be observed if all physical activity was performed outdoors.

## Conclusion

The current study is the first to show that higher levels of temperature and air pollution indices, even in locations with relatively good AQI and moderate temperatures, contribute to lower MVPA and higher sedentary time in the context of MBS. Less marked decreases in MVPA at similar higher temperatures after MBS compared to before MBS suggests patients may have increased heat tolerance during MVPA although confirmation of this finding and potential mechanism is needed. Weather and environmental factors should be considered in prescriptions and strategies to promote more active lifestyles among adults who have undergone MBS.

## Supplementary Material

Supplementary Material

## Data Availability

The data that support the findings of this study are openly available at the following URL/DOI: https://osf.io/34usq/.
