# Supplementary material for: Associations of weather and air pollution with objective physical activity and sedentary time before and after bariatric surgery: a secondary analysis of a prospective cohort study
Source: Environ Res Commun. Author manuscript; Available in PMC 2024 Oct 28. (PMC11514705; doi:10.1088/2515-7620/ad64b2)
Supplement: Supplementary Material [file NIHMS2016375-supplement-Supplementary_Material.pdf]

Supplemental table 1: STROBE Statement—checklist of items that should be included in reports of observational studies

	Item No	Recommendation	Page No
**Title and abstract**	1	(*a*) Indicate the study's design with a commonly used term in the title or the abstract	4
		(*b*) Provide in the abstract an informative and balanced summary of what was done and what was found	4
**Introduction**			
Background/rationale	2	Explain the scientific background and rationale for the investigation being reported	5-7
Objectives	3	State specific objectives, including any prespecified hypotheses	7
**Methods**			
Study design	4	Present key elements of study design early in the paper	8-9
Setting	5	Describe the setting, locations, and relevant dates, including periods of recruitment, exposure, follow-up, and data collection	9
Participants	6	(*a*) *Cohort study*—Give the eligibility criteria, and the sources and methods of selection of participants. Describe methods of follow-up<br>*Case-control study*—Give the eligibility criteria, and the sources and methods of case ascertainment and control selection. Give the rationale for the choice of cases and controls<br>*Cross-sectional study*—Give the eligibility criteria, and the sources and methods of selection of participants	8
		(*b*) *Cohort study*—For matched studies, give matching criteria and number of exposed and unexposed<br>*Case-control study*—For matched studies, give matching criteria and the number of controls per case	
Variables	7	Clearly define all outcomes, exposures, predictors, potential confounders, and effect modifiers. Give diagnostic criteria, if applicable	9-10
Data sources/ measurement	8*	For each variable of interest, give sources of data and details of methods of assessment (measurement). Describe comparability of assessment methods if there is more than one group	9-10
Bias	9	Describe any efforts to address potential sources of bias	9-10
Study size	10	Explain how the study size was arrived at	8

Quantitative variables	11	Explain how quantitative variables were handled in the analyses. If applicable, describe which groupings were chosen and why	9-10
Statistical methods	12	(*a*) Describe all statistical methods, including those used to control for confounding	11,12
		(*b*) Describe any methods used to examine subgroups and interactions	11,12
		(*c*) Explain how missing data were addressed	11,12
		(*d*) *Cohort study*—If applicable, explain how loss to follow-up was addressed<br>*Case-control study*—If applicable, explain how matching of cases and controls was addressed<br>*Cross-sectional study*—If applicable, describe analytical methods taking account of sampling strategy	11,12
		(*e*) Describe any sensitivity analyses	12

Results

Participants	13*	(a) Report numbers of individuals at each stage of study—eg numbers potentially eligible, examined for eligibility, confirmed eligible, included in the study, completing follow-up, and analysed	8
		(b) Give reasons for non-participation at each stage	
		(c) Consider use of a flow diagram	Suppl Fig 1
Descriptive data	14*	(a) Give characteristics of study participants (eg demographic, clinical, social) and information on exposures and potential confounders	Suppl Table 1
		(b) Indicate number of participants with missing data for each variable of interest	8, Flow diag
		(c) *Cohort study*—Summarise follow-up time (eg, average and total amount)	
Outcome data	15*	*Cohort study*—Report numbers of outcome events or summary measures over time	8
		Case-control study—Report numbers in each exposure category, or summary measures of exposure	

		Cross-sectional study—Report numbers of outcome events or summary measures	
Main results	16	(*a*) Give unadjusted estimates and, if applicable, confounder-adjusted estimates and their precision (eg, 95% confidence interval). Make clear which confounders were adjusted for and why they were included	12-14
		(*b*) Report category boundaries when continuous variables were categorized	
		(*c*) If relevant, consider translating estimates of relative risk into absolute risk for a meaningful time period	
Other analyses	17	Report other analyses done—eg analyses of subgroups and interactions, and sensitivity analyses	12-14
**Discussion**			
Key results	18	Summarise key results with reference to study objectives	14-15
Limitations	19	Discuss limitations of the study, taking into account sources of potential bias or imprecision. Discuss both direction and magnitude of any potential bias	18
Interpretation	20	Give a cautious overall interpretation of results considering objectives, limitations, multiplicity of analyses, results from similar studies, and other relevant evidence	14-18
Generalisability	21	Discuss the generalisability (external validity) of the study results	18
**Other information**			
Funding	22	Give the source of funding and the role of the funders for the present study and, if applicable, for the original study on which the present article is based	1

*Give information separately for cases and controls in case-control studies and, if applicable, for exposed and unexposed groups in cohort and cross-sectional studies.

Note: An Explanation and Elaboration article discusses each checklist item and gives methodological background and published examples of transparent reporting. The STROBE checklist is best used in conjunction with this article (freely available on the Web sites of PLoS Medicine at http://www.plosmedicine.org/, Annals of Internal Medicine at http://www.annals.org/, and Epidemiology at http://www.epidem.com/). Information on the STROBE Initiative is available at www.strobe-statement.org

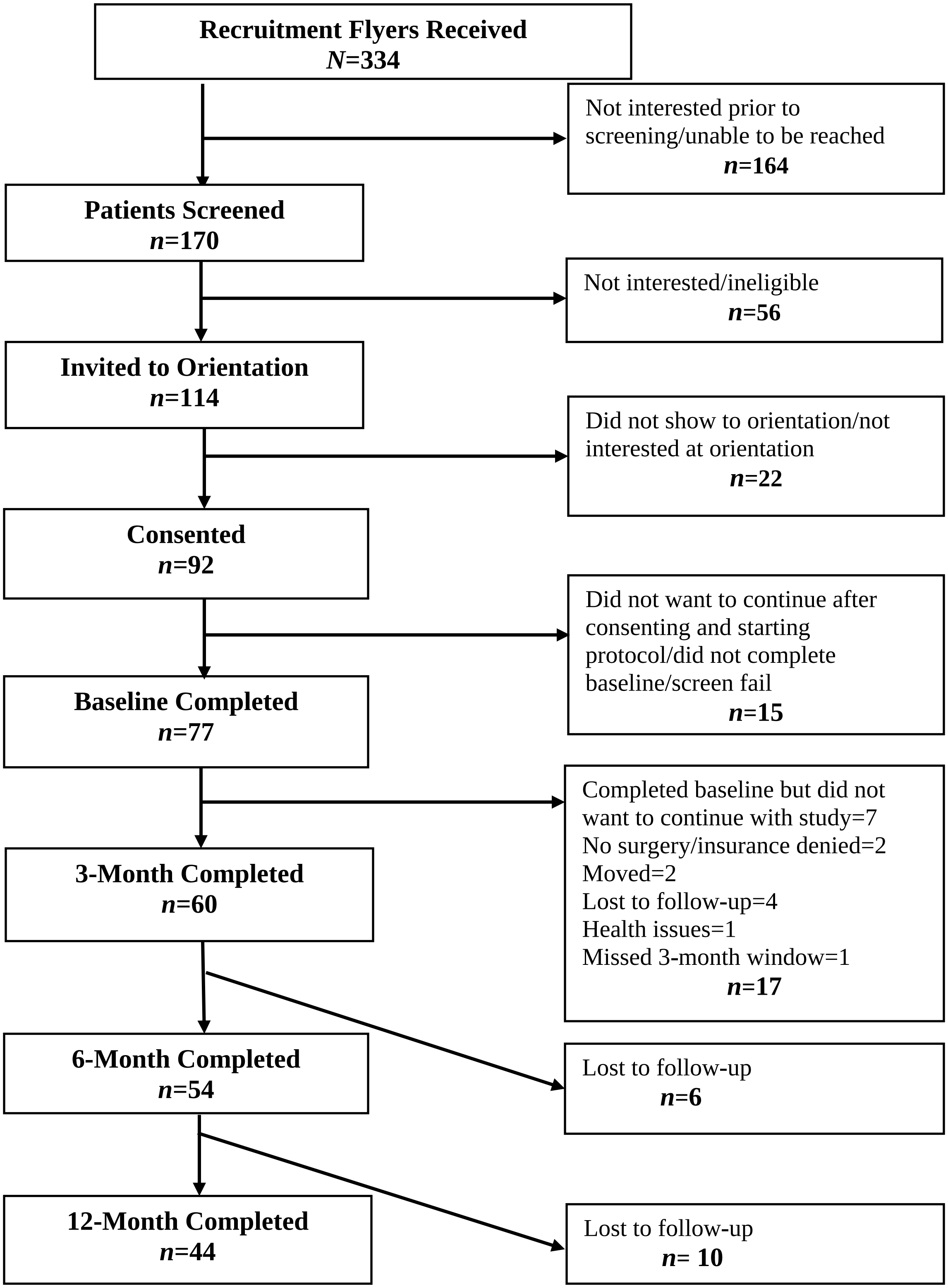

Supplemental Figure 1: Flow Chart

Supplemental Table 2. Accelerometer data (LPA, MVPA, sedentary time) according to the 4 time of assessments and seasons

	**Before MBS N = 826 mean (SD)**	**3 months after MBS N=683 mean (SD)**	**6 months after MBS N=603 mean (SD)**	**12 months after MBS N=465 mean (SD)**	**p.overall**
**Body mass index** (kg.m^2)	45.9 (7.55)	42.5 (9.03)	35.0 (6.00)	33.4 (6.02)	<0.001
**LPA** (min/d)	343 (104)	330 (121)	353 (110)	346 (113)	0.003
**MVPA** (min/d)	37.7 (31.4)	42.2 (45.5)	44.1 (41.3)	43.3 (54.8)	0.020
**Sedentary time** (min/d)	640 (189)	627 (176)	604 (178)	587 (156)	<0.001
**Seasons**	**N %**	**N %**	**N %**	**N %**	<0.001
Autumn	192 (23.2%)	177 (25.9%)	163 (27.0%)	156 (33.5%)	
Spring	227 (27.5%)	146 (21.4%)	142 (23.5%)	120 (25.8%)	
Summer	221 (26.8%)	243 (35.6%)	125 (20.7%)	80 (17.2%)	
Winter	186 (22.5%)	117 (17.1%)	173 (28.7%)	109 (23.4%)	
	**Spring N=688** N %	**Summer N=635** N %	**Fall N=669** N %	**Winter N=585** N %	**p.overall**
**LPA** (min/d)	353 (107)	331 (124)	349 (110)	337 (104)	0.001
**MVPA** (min/d)	45.3 (49.9)	41.3 (41.6)	37.2 (31.0)	41.6 (45.1)	0.006
**Sedentary time** (min/d)	601 (180)	620 (170)	625 (178)	630 (183)	0.016

N = number of observations; LPA = light intensity physical activity; MVPA = moderate-to-vigorous intensity physical activity.

Supplemental Table 3. Associations of weather conditions and air pollution with MVPA

Models: Associations with MVPA	Averages temperature (°C)<br>β [95%CI]	Maximal temperature (°C)<br>β [95%CI]	WBGT (°F)<br>β [95%CI]	Air Quality Index<br>β [95%CI]
Number of days	-0.02 [-0.02, -0.02] ***	-0.02 [-0.02, -0.02] ***	-0.02 [-0.02, -0.02] ***	-0.02 [-0.02, -0.02] ***
Weekends	-0.07 [-0.08, -0.06] ***	-0.07 [-0.09, -0.06] ***	-0.07 [-0.09, -0.06] ***	-0.07 [-0.09, -0.06] ***
Season: spring	0.09 [-0.007, 0.2] +	0.1 [0.03, 0.2] **	0.1 [0.008, 0.2] *	0.1 [0.03, 0.2] **
Season: summer	0.007 [-0.1, 0.1]	0.03 [-0.09, 0.1]	0.01 [-0.1, 0.1]	-0.002 [-0.1, 0.1]
Season: winter	-0.07 [-0.1, -0.0003] *	-0.06 [-0.1, 0.01]	-0.1 [-0.2, -0.06] ***	-0.05 [-0.1, 0.02]
Sex: Males	-0.5 [-0.8, -0.2] ***	-0.5 [-0.8, -0.2] ***	-0.5 [-0.8, -0.2] ***	-0.5 [-0.8, -0.2] ***
Body mass index (kg.m^2)	-0.01 [-0.02, -0.0009] *	-0.01 [-0.02, -0.001] *	-0.01 [-0.02, -0.001] *	-0.01 [-0.02, -0.0008] *
Age	-0.01 [-0.02, -0.005] **	-0.01 [-0.02, -0.006] **	-0.01 [-0.02, -0.006] **	-0.01 [-0.02, -0.006] ***
lag_MVPA (min)	-0.00005 [-0.0002, 0.0001]	0.000008 [-0.0001, 0.0002]	0.00002 [-0.0001, 0.0002]	-0.000005 [-0.0002, 0.0001]
s (Snow (cm))	***	***	***	***
s (Rain precipitation (cm))	***	***	***	***
lag_ Averaged temperature (Celsius)	-0.002 [-0.004, 0.0003] +			
**s (Averaged temperature (Celsius))**	***			
lag_(Maximal Temperature (Celsius))		0.001 [-0.0004, 0.003]		
**s (Maximal Temperature (Celsius))**		***		
lag_WBGT_min			-0.002 [-0.003, -0.0006] **	

Supplemental Table 3. Associations of weather conditions and air pollution with MVPA

**s WBGT**			***	
lag_ Air Quality Index				-0.0001 [-0.0006, 0.0004]
**s (Air Quality Index)**				***
N (observation number)	2252	2252	2214	2252
R^2	**0.642**	**0.643**	**0.630**	**0.638**
AIC	31842.5	31822.8	31381.2	31994.1

* = ≤.05; ** =≤.01; *** =≤.001; MVPA = moderate-to-vigorous intensity physical activity; s = smoothed; WBGT = Wet Bulb Globe Temperature.

Supplemental Table 4. Associations of weather conditions and air pollution with light intensity physical activity

Models: Associations with LPA	Averages temperature (°C)<br>β [95%CI]	Maximal temperature (°C)<br>β [95%CI]	WBGT (°F)<br>β [95%CI]	Air Quality Index<br>β [95%CI]
Number of days	-4.5 [-5.6, -3.3] ***	-4.5 [-5.6, -3.3] ***	-4.4 [-5.6, -3.3] ***	-4.4 [-5.5, -3.3] ***
Weekends	-28.4 [-36.2, -20.6] ***	-28.3 [-36.0, -20.5] ***	-27.8 [-35.7, -20.0] ***	-28.8 [-36.6, -21.1] ***
Season: spring	-9.3 [-31.5, 12.8]	-8.8 [-30.9, 13.4]	-10.0 [-32.3, 12.4]	-11.7 [-33.5, 10.1]
Season: summer	-2.4 [-26.0, 21.2]	-3.6 [-26.6, 19.4]	-3.3 [-26.5, 19.9]	-2.1 [-23.9, 19.7]
Season: winter	-5.7 [-29.4, 18.1]	-6.9 [-30.2, 16.3]	-7.5 [-31.4, 16.4]	-12.4 [-34.3, 9.4]
Sex: Males	-40.1 [-64.8, -15.4] **	-39.8 [-64.5, -15.1] **	-40.5 [-65.3, -15.8] **	-40.5 [-65.4, -15.7] **
Body mass index (kg.m^2)	-1.0 [-1.9, -0.05] *	-1.0 [-1.9, -0.05] *	-1.0 [-1.9, -0.07] *	-0.8 [-1.8, 0.1] +
Age	0.1 [-0.6, 0.8]	0.1 [-0.6, 0.8]	0.08 [-0.7, 0.8]	0.1 [-0.6, 0.9]
lag_LPA (min)	0.2 [0.1, 0.2] ***	0.2 [0.1, 0.2] ***	0.2 [0.1, 0.2] ***	0.2 [0.1, 0.2] ***
s (Snow (cm))	*	*	*	
s (Rain precipitation (cm))	NS	NS	NS	
Rain precipitation (cm)				-9.4 [-21.3, 2.6]
Snow (cm)				-5.8 [-10.5, -1.1] *
lag_ Averaged temperature (Celsius)	-0.1 [-1.2, 0.9]			
**s (Averaged temperature (Celsius))**	**NS**			
lag_(Maximal Temperature (Celsius))		0.04 [-0.8, 0.8]		

Supplemental Table 4. Associations of weather conditions and air pollution with light intensity physical activity

**s (Maximal Temperature (Celsius))**		**NS**		
lag_WBGT_min			00.2 [-0.4, 0.8]	
**s WBGT**			**NS**	
lag_ Air Quality Index				0.07 [-0.2, 0.3]
**Air Quality Index**				-0.2 [-0.5, 0.04]
N (observation number)	2261	2261	2223	2261
R^2	0.424	0.424	0.422	0.136
AIC	26708.3	26705.6	26272.9	26851.5

* = ≤.05; ** =≤.01; *** =≤.001; LPA = light intensity physical activity; s = smoothed; WBGT = Wet Bulb Globe Temperature.

Supplemental Table 5. Associations of weather conditions and air pollution with sedentary time

Models: Associations with sedentary time	Averages temperature (°C)<br>β [95%CI]	Maximal temperature (°C)<br>β [95%CI]	WBGT (°F)<br>β [95%CI]	Air Quality Index<br>β [95%CI]
Number of days	-0.8 [-2.6, 1.0]	-0.8 [-2.6, 1.0]	-0.9 [-2.7, 1.0]	-0.8 [-2.7, 1.0]
Weekends	-14.4 [-27.0, -1.8] *	-14.2 [-26.7, -1.6] *	-16.1 [-28.8, -3.4] *	-13.5 [-26.0, -1.0] *
Season: spring	9.1 [-26.8, 45.1]	9.0 [-26.9, 44.8]	11.0 [-25.3, 47.3]	12.0 [-23.4, 47.5]
Season: summer	13.8 [-23.9, 51.4]	11.4 [-25.6, 48.4]	15.6 [-21.7, 52.9]	15.7 [-19.6, 51.0]
Season: winter	8.7 [-29.5, 46.8]	9.7 [-27.7, 47.2]	12.9 [-25.6, 51.4]	13.3 [-22.1, 48.7]
Sex: Males	31.6 [-8.3, 71.4]	31.3 [-8.4, 71.1]	30.7 [-9.4, 70.7]	30.8 [-9.0, 70.6]
Body mass index (kg.m^2)	3.4 [1.9, 4.9] ***	3.4 [1.9, 4.9] ***	3.5 [1.9, 5.0] ***	3.4 [1.9, 4.9] ***
Age	2.6 [1.4, 3.8] ***	2.6 [1.4, 3.8] ***	2.5 [1.4, 3.7] ***	2.6 [1.4, 3.8] ***
lag_sedantary time (min)	0.09 [0.05, 0.1] ***	0.09 [0.05, 0.1] ***	0.09 [0.05, 0.1] ***	0.09 [0.05, 0.1] ***
s (Snow (cm))	*	*	*	*
s (Rain precipitation (cm))	NS	NS	NS	NS
lag_ Averaged temperature (Celsius)	-0.7 [-2.4, 1.0]			
**<u>s (Averaged temperature (Celsius))</u>**	**NS**			
lag_(Maximal Temperature (Celsius))		-0.7 [-2.0, 0.6]		
**<u>s (Maximal Temperature (Celsius))</u>**		**NS**		
lag_WBGT_min			-0.4 [-1.3, 0.6]	

Supplemental Table 5. Associations of weather conditions and air pollution with sedentary time

**s WBGT**			**NS**	
lag_ Air Quality Index				-0.4 [-0.8, 0.001]
**s (Air Quality Index)**				**NS**
N (observation number)	2252	2252	2214	2252
R^2	0.393	0.394	0.394	0.394
AIC	28758.2	28757.0	28278.6	28752.3

* = ≤.05; ** =≤.01; *** =≤.001; MVPA = moderate-to-vigorous intensity physical activity; s = smoothed; WBGT = Wet Bulb Globe Temperature.

Supplemental Table 6. R results for the sensitivity analysis for associations of weather conditions and air pollution with moderate-to-vigorous intensity physical activity before and after metabolic and bariatric surgery

Models: Associations with MVPA	Averages temperature (°C)<br>β [95%CI]	Maximal temperature (°C)<br>β [95%CI]	WBGT (°F)<br>β [95%CI]	Air Quality Index<br>β [95%CI]
Number of days	-0.02 [-0.02, -0.02] ***	-0.02 [-0.02, -0.02] ***	-0.02 [-0.02, -0.02] ***	-0.02 [-0.02, -0.02] ***
Weekends	-0.07 [-0.09, -0.06] ***	-0.07 [-0.09, -0.06] ***	-0.07 [-0.09, -0.06] ***	-0.07 [-0.08, -0.05] ***
Season: spring	0.1 [0.02, 0.2] *	0.1 [0.04, 0.2] **	0.1 [0.02, 0.2] *	0.1 [0.05, 0.2] **
Season: summer	0.03 [-0.09, 0.2]	0.04 [-0.08, 0.2]	0.01 [-0.1, 0.1]	0.006 [-0.1, 0.1]
Season: winter	-0.08 [-0.2, -0.01] *	-0.07 [-0.1, 0.003] +	-0.2 [-0.2, -0.08] ***	-0.05 [-0.1, 0.02]
Sex: Males	-0.5 [-0.8, -0.2] ***	-0.5 [-0.8, -0.2] ***	-0.5 [-0.8, -0.2] ***	-0.5 [-0.8, -0.2] ***
Body mass index (kg.m^2)	-0.01 [-0.02, -0.0008] *	-0.01 [-0.02, -0.0007] *	-0.01 [-0.02, -0.001] *	-0.01 [-0.02, -0.0006] *
Age	-0.01 [-0.02, -0.005] **	-0.01 [-0.02, -0.006] **	-0.01 [-0.02, -0.006] **	-0.01 [-0.02, -0.006] **
lag_MVPA (min)	-0.00006 [-0.0002, 0.00008]	-0.00001 [-0.0002, 0.0001]	-0.00004 [-0.0002, 0.0001]	0.000003 [-0.0001, 0.0001]
s (Snow (cm))	***	***	***	***
s (Rain precipitation (cm))	***	***	***	***
lag_ Averaged temperature (Celsius)	-0.003 [-0.005, -0.001] **			
**s (Averaged temperature (Celsius)) before surgery**	*******			
**s (Averaged temperature (Celsius)) after surgery**	*******			
lag_(Maximal Temperature (Celsius))		0.001 [-0.0004, 0.003]		

Supplemental Table 6. R results for the sensitivity analysis for associations of weather conditions and air pollution with moderate-to-vigorous intensity physical activity before and after metabolic and bariatric surgery

**s (Maximal Temperature (Celsius)) before surgery**		***		
**s (Maximal Temperature (Celsius)) after surgery**		***		
lag_WBGT_min			-0.002 [-0.003, -0.0009] ***	
**s WBGT before surgery**			***	
**s WBGT after surgery**			***	
lag_ Air Quality Index				-0.0003 [-0.0008, 0.0002]
**s (Air Quality Index) before surgery**				***
**s (Air Quality Index) after surgery**				***
N (observation number)	2252	2252	2214	2252
R^2	0.649	0.647	0.638	0.639
AIC	31542.0	31641.6	31095.9	31915.3

* = ≤.05; ** =≤.01; *** =≤.001; MVPA = moderate-to-vigorous intensity physical activity; s = smoothed; WBGT = Wet Bulb Globe Temperature.

Supplemental Table 7. R results for the sensitivity analysis for associations of weather conditions and air pollution with light intensity physical activity before and after metabolic and bariatric surgery

Models: Associations with LPA	Averages temperature (°C) β [95%CI]	Maximal temperature (°C) β [95%CI]	WBGT (°F) β [95%CI]	Air Quality Index before surgery β [95%CI]	Air Quality Index after surgery β [95%CI]
Number of days	-4.5 [-5.6, -3.4] ***	-4.5 [-5.6, -3.4] ***	-4.5 [-5.6, -3.4] ***	-2.2 [-4.1, -0.4] *	-5.5 [-6.9, -4.1] ***
Weekends	-28.0 [-35.8, -20.3] ***	-28.0 [-35.7, -20.2] ***	-27.7 [-35.6, -19.8] ***	-30.9 [-44.2, -17.6] ***	-28.4 [-37.9, -18.9] ***
Season: spring	-10.2 [-32.5, 12.1]	-9.7 [-32.0, 12.6]	-10.5 [-32.8, 11.8]	-7.5 [-43.3, 28.4]	-21.3 [-49.2, 6.6]
Season: summer	-3.5 [-27.0, 20.1]	-4.6 [-27.6, 18.4]	-4.0 [-27.0, 19.1]	-10.2 [-49.1, 28.8]	-2.7 [-29.5, 24.1]
Season: winter	-6.7 [-30.5, 17.0]	-7.0 [-30.2, 16.3]	-8.2 [-32.0, 15.6]	-21.4 [-59.0, 16.1]	-11.7 [-39.1, 15.8]
Sex: Males	-39.8 [-64.7, -14.9] **	-40.2 [-65.1, -15.4] **	-39.4 [-64.2, -14.7] **	-57.8 [-102.4, -13.2] *	-36.8 [-67.5, -6.1] *
Body mass index (kg.m^2)	-1.0 [-1.9, -0.05] *	-1.0 [-1.9, -0.03] *	-1.0 [-1.9, -0.07] *	-1.5 [-3.4, 0.4]	-0.8 [-2.1, 0.5]
Age	0.1 [-0.6, 0.9]	0.1 [-0.6, 0.9]	0.1 [-0.6, 0.8]	0.03 [-1.3, 1.3]	0.3 [-0.6, 1.2]
lag_LPA (min)	0.2 [0.1, 0.2] ***	0.2 [0.1, 0.2] ***	0.2 [0.1, 0.2] ***	0.2 [0.08, 0.2] ***	0.2 [0.1, 0.2] ***
s (Snow (cm))	*	*	*		
s (Rain precipitation (cm))	***	***	***		
Rain precipitation (cm)				-5.1 [-25.5, 15.3]	-10.7 [-25.6, 4.1]
Snow (cm)				-6.1 [-13.5, 1.4]	-6.0 [-12.0, -0.04] *
lag_ Averaged temperature (Celsius)	-0.2 [-1.2, 0.9]				
**s (Averaged temperature (Celsius)) before surgery**	**NS**				

Supplemental Table 7. R results for the sensitivity analysis for associations of weather conditions and air pollution with light intensity physical activity before and after metabolic and bariatric surgery

**s (Averaged temperature (Celsius)) after surgery**	**NS**				
lag_(Maximal Temperature (Celsius))		0.05 [-0.8, 0.8]			
**s (Maximal Temperature (Celsius)) before surgery**		**NS**			
**s (Maximal Temperature (Celsius)) after surgery**		**NS**			
lag_WBGT_min			0.2 [-0.4, 0.8]		
**s WBGT before surgery**			**NS**		
**s WBGT after surgery**			**NS**		
lag_ Air Quality Index				0.2 [-0.2, 0.6]	-0.03 [-0.4, 0.3]
**Air Quality Index**				-0.3 [-0.7, 0.1]	-0.1 [-0.5, 0.2]
N (observation number)	2261	2223	2261	717	1544
R^2	0.426	0.421	0.425	0.147	0.143
AIC	26706.1	26275.7	26705.3	8425.1	18385.5

* = ≤.05; ** =≤.01; *** =≤.001; LPA = light intensity physical activity; s = smoothed; WBGT = Wet Bulb Globe Temperature.

Supplemental Table 8. R results for the sensitivity analysis for associations of weather conditions and air pollution with sedentary time before and after metabolic and bariatric surgery

Models: Associations with sedentary time	Averages temperature (°C)<br>β [95%CI]	Maximal temperature (°C)<br>β [95%CI]	WBGT (°F)<br>β [95%CI]	Air Quality Index<br>β [95%CI]
Number of days	-0.8 [-2.6, 1.0]	-0.8 [-2.6, 1.0]	-0.8 [-2.7, 1.0]	-0.8 [-2.6, 1.0]
Weekends	-14.6 [-27.2, -2.0] *	-14.3 [-26.9, -1.8] *	-16.4 [-29.1, -3.7] *	-13.0 [-25.5, -0.5] *
Season: spring	9.9 [-26.1, 45.8]	10.0 [-25.9, 45.8]	11.7 [-24.7, 48.0]	12.5 [-23.0, 48.0]
Season: summer	15.0 [-22.3, 52.3]	12.9 [-23.9, 49.6]	14.7 [-22.4, 51.8]	15.2 [-20.1, 50.5]
Season: winter	8.2 [-29.9, 46.2]	9.6 [-27.8, 46.9]	12.4 [-26.1, 50.8]	12.5 [-23.0, 47.9]
Sex: Males	31.7 [-8.2, 71.6]	32.3 [-7.5, 72.1]	30.5 [-9.6, 70.5]	31.4 [-8.4, 71.3]
Body mass index (kg.m^2)	3.5 [2.0, 5.0] ***	3.5 [1.9, 5.0] ***	3.5 [2.0, 5.0] ***	3.4 [1.9, 5.0] ***
Age	2.5 [1.3, 3.7] ***	2.5 [1.4, 3.7] ***	2.5 [1.3, 3.7] ***	2.6 [1.4, 3.8] ***
lag_sedantary time (min)	0.09 [0.05, 0.1] ***	0.09 [0.05, 0.1] ***	0.09 [0.05, 0.1] ***	0.09 [0.05, 0.1] ***
s (Snow (cm))	*	*	*	*
s (Rain precipitation (cm))	NS	NS	NS	NS
lag_ Averaged temperature (Celsius)	-0.7 [-2.4, 1.0]			
**s (Averaged temperature (Celsius)) before surgery**	**NS**			
**s (Averaged temperature (Celsius)) after surgery**	**NS**			
lag_(Maximal Temperature (Celsius))		-0.7 [-2.0, 0.6]		

Supplemental Table 8. R results for the sensitivity analysis for associations of weather conditions and air pollution with sedentary time before and after metabolic and bariatric surgery

**s (Maximal Temperature (Celsius)) before surgery**		**NS**		
**s (Maximal Temperature (Celsius)) after surgery**		**NS**		
lag_WBGT_min			-0.4 [-1.3, 0.6]	
**s WBGT before surgery**			**NS**	
**s WBGT after surgery**			**NS**	
lag_ Air Quality Index				-0.4 [-0.8, 0.02]
**s (Air Quality Index) before surgery**				*****
**s (Air Quality Index) after surgery**				**NS**
N (observation number)	2252	2214	2252	2252
R^2	0.394	0.395	0.394	0.395
AIC	28756.7	28273.2	28756.2	28750.9

* = ≤.05; ** =≤.01; *** =≤.001; MVPA = moderate-to-vigorous intensity physical activity; s = smoothed; WBGT = Wet Bulb Globe Temperature.

Supplemental Figure 2: Associations of weather indices (average temperature, maximal temperature, WBGT) and air pollution (Air quality index) with moderate-vigorous intensity physical activity.

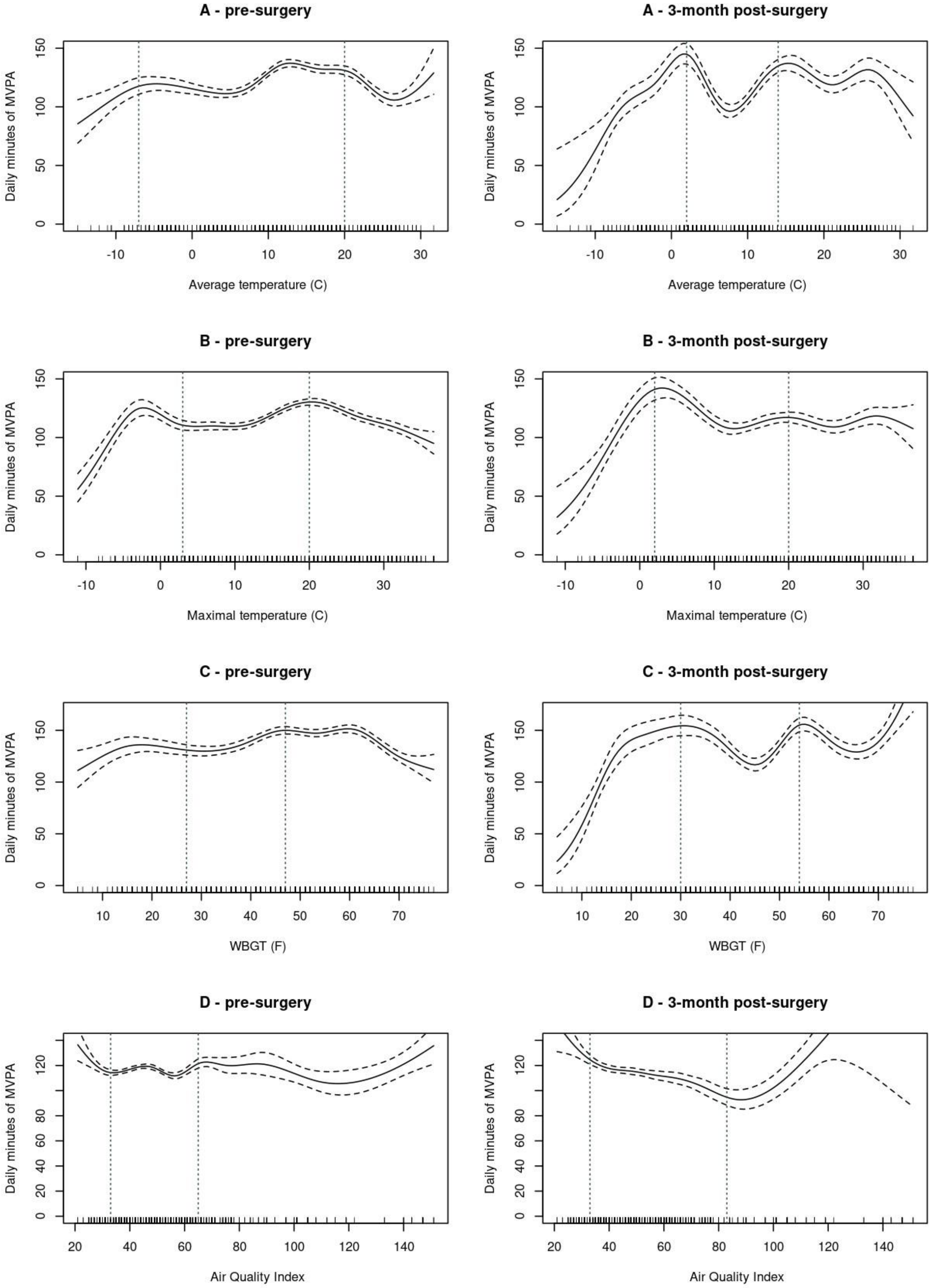

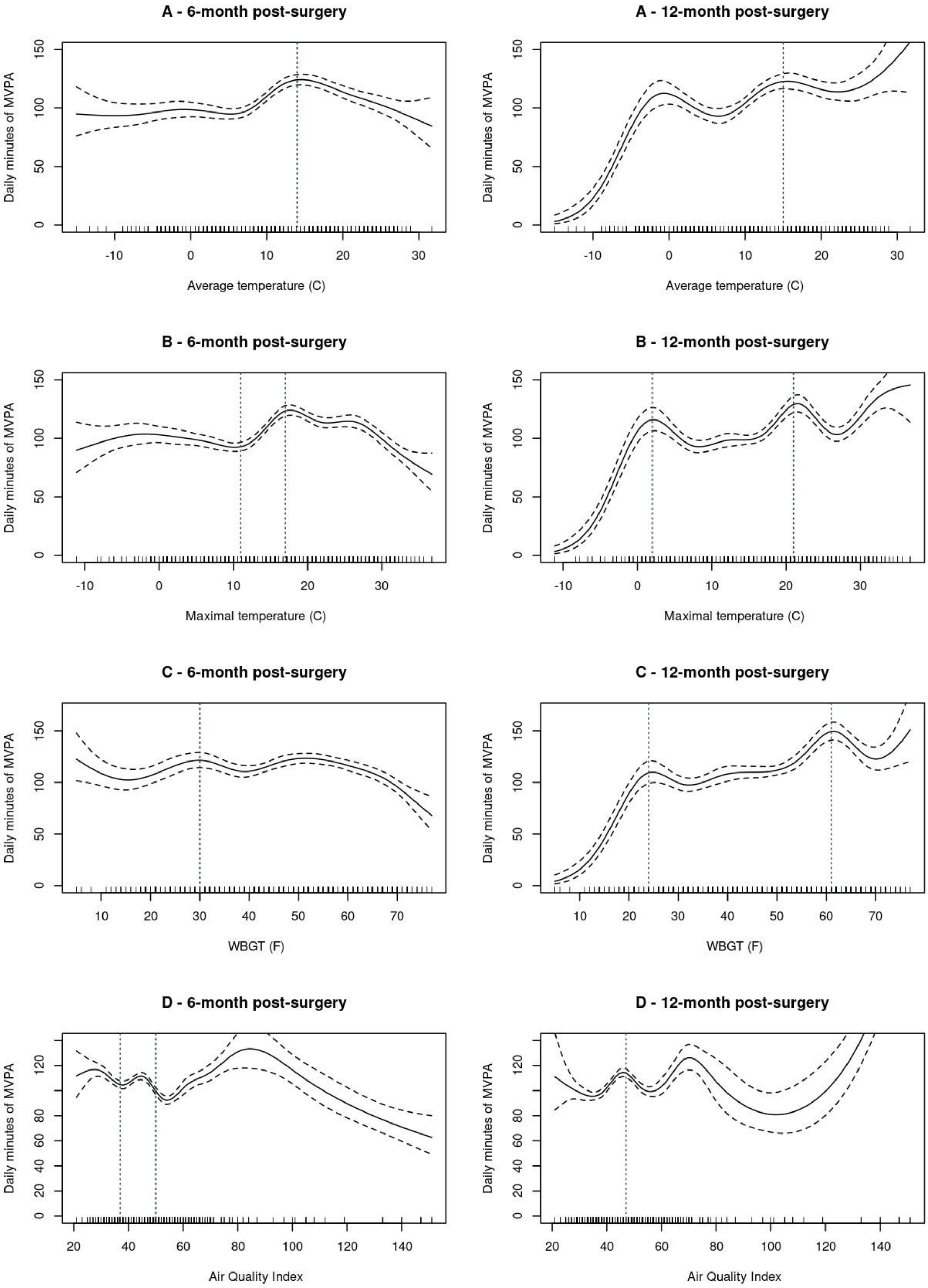
A - 6-month post-surgery
A - 12-month post-surgery
Daily minutes of MVPA
Average temperature (C)
B - 6-month post-surgery
B - 12-month post-surgery
Daily minutes of MVPA
Maximal temperature (C)
C - 6-month post-surgery
C - 12-month post-surgery
Daily minutes of MVPA
WBGT (F)
D - 6-month post-surgery
D - 12-month post-surgery
Daily minutes of MVPA
Air Quality Index

Note: The lines show the smoothed function from GAM for daily minutes of MVPA, and the shaded area indicates the 95% confidence interval. Each model is adjusted for age, body mass index, gender, days number, season, weekend day (Yes/No), precipitation, snowfall, lag-1 of MVPA and weather variables. Vertical lines are placed based on visual observation.
